# Response of Landscape Types and Shorebird Diversity to Extreme Drought Climate in Poyang Lake, China During the Non-Breeding Period

**DOI:** 10.3390/ani15101399

**Published:** 2025-05-12

**Authors:** Zhongshan Yan, Mingqin Shao

**Affiliations:** College of Life Science, Jiangxi Normal University, Nanchang 330022, China; 202340100664@jxnu.edu.cn

**Keywords:** landscape type, extreme climate, shorebirds, Poyang Lake, wetland

## Abstract

Habitat use by shorebirds is described in Poyang Lake in the Nanji Wetland National Nature Reserve (“Nanji Wetland”) and the Wuxing reclamation region (“Wuxing”) during the non-breeding periods of 2022 (extreme drought year) and 2023 (normal water year), using the sample point method. The results indicated that the deep water area in the extreme drought year at Nanji Wetland and Wuxing was smaller than in the normal water year, while the mudflat area was larger. Grassland area during the early and middle parts of the extreme drought year was lower than in the normal water year, and fluctuations in shallow water area were relatively small in both regions. Landscape indices at Nanji Wetland exhibited greater variability, with most indices being lower in the extreme drought year. Most landscape indices in Wuxing were consistent across years. The number of species and individuals was higher in the extreme drought year than in the normal water year at Nanji Wetland, whereas the opposite trend was observed at Wuxing, suggesting that the large, protected area served as a refuge for many shorebirds. The mudflat area was found to be strongly and positively correlated with the total number of shorebirds, the number of species, and the populations of *Vanellus vanellus* and *Tringa erythropus*. The SHDI was found to exhibit a strong negative correlation with the number of shorebird species and the populations of *Limosa limosa*. The results indicate that the mudflat area is critical for maintaining shorebird diversity in Poyang Lake, and reducing the SHDI may enhance shorebird diversity. Our findings have to be further tested for long-term period in the future. These findings provide guidelines for shorebird population conservation and habitat management strategies.

## Simple Summary

Habitat use by shorebirds is described in Poyang Lake in the Nanji Wetland National Nature Reserve (“Nanji Wetland”) and the Wuxing reclamation region (“Wuxing”) during the non-breeding periods of 2022 (extreme drought year) and 2023 (normal water year), using the sample point method. The results showed that (1) the area of deep water decreased and the area of mudflats expanded in the extreme drought year, and the area of grassland decreased significantly during the early and middle periods, while the fluctuation of shallow water was less pronounced; (2) the landscape indices of Nanji Wetland generally declined in the extreme drought year, and there was no obvious difference in Wuxing; (3) in terms of species diversity, the number of species and individuals in Nanji Wetland during the extreme drought year were greater than that in the normal water year, whereas Wuxing showed the opposite trend, indicating that the larger protected area could serve as a refuge under extreme climate conditions; (4) in correlation analyses, the mudflat area was found to be strongly and positively correlated with the total number of shorebirds, the number of species, and key populations (such as *Vanellus vanellus* and *Tringa erythropus*), whereas the diversity index (SHDI) exhibited a negative correlation with some species. This study confirms that maintaining mudflat habitats and landscape connectivity is a key strategy to safeguard shorebird diversity. Our findings have to be further tested for long-term period in the future. Our results can provide a scientific basis for ecological management.

## 1. Introduction

Wetland landscape patterns refer to the spatial arrangement and composition of landscape patches of various sizes and shapes in wetlands [1], resulting from the combined influence of natural and socio-economic conditions. Influenced by climate change and human activities, the area of natural landscapes has significantly decreased, and the shape of patches has become simpler and more regular, accompanied by severe fragmentation [2,3]. The wetland landscape pattern is a key factor influencing waterbird behavior and the distribution of foraging areas [4]. The landscape composition, configuration, and function significantly affect waterbird richness and abundance. Good connectivity between landscapes aids waterbirds in finding suitable stopover and foraging sites during migration. Additionally, complexly shaped habitat patches typically have more edges and provide diverse ecological niches for waterbirds, all contributing to increased waterbird diversity [5]. The area and distribution of each landscape type determine waterbird food abundance and influence the degree of wetland utilization by waterbirds [6,7]. Quantitative studies on waterbird diversity and wetland landscape patterns help understand the ecological needs of waterbirds and their responses to changes in landscape patterns, providing a scientific basis for the development of conservation measures for birds and their habitats.

Shorebirds primarily inhabit wetland environments such as water areas, marshes, and sandy beaches [8,9]. Most shorebirds are migratory birds, highly dependent on wetlands during migration and extremely sensitive to changes in water body area, making them an indicator species for the health of wetland ecosystems [10]. Climatic changes alter the migratory routes and food abundance of shorebirds [11]. Increased vegetation cover reduces the area of shorebirds’ roosting and feeding grounds. Diverse food species play an important role in maintaining shorebird diversity [12]. An increased predation risk threatens their survival and reproduction [13], and habitat loss and degradation compress the shorebirds’ living space, altering their ecological niche [14,15]. A historically rare drought occurred in the Yangtze River Basin in 2022, affecting the wetland of Poyang Lake [16]. The water level of Poyang Lake dropped dramatically due to persistent high temperatures and low rainfall, resulting in the worst drought conditions since 1949. The water area of the lake was about only 560 km^2^, about one-fifth of the area in a normal water year. The drought caused large-scale drying of the lake bed and cracking of the soil at the lake bottom, creating a vast area of “grassland” [17]. Our main hypothesis is that the mudflat area in an extreme drought year is larger than in a normal water year, while the shallow water area remains relatively unchanged. This will lead to an increase in shorebird species richness and total numbers in extreme drought year in the Nanji Wetland due to the larger area and higher capacity, while a decline in numbers will occur in Wuxing with lesser natural habitat area. Lower water levels in the Nanji Wetland will result in an increase in the mudflat area. The shift from deep water to shallow water areas maintains the proportion of shallow water areas, and the habitat advantage for shorebirds in utilizing shallow water is preserved. Therefore, during an extreme drought year, the Nanji Wetland supports shorebird populations to a greater extent, resulting in increased abundance and population numbers. On the other hand, due to the limited lake area and extensive cropland, the shorebird population decreased significantly when the lake lost its natural habitat advantages during extreme drought. To produce those findings, this study focused on the Nanji Wetland National Nature Reserve in Poyang Lake, Jiangxi Province, and the Wuxing reclamation region, monitoring shorebirds during the autumn migration and overwintering periods in extreme drought and a normal water year. The objectives were (1) to accumulate basic data on shorebirds in the two areas and assess the importance of these areas for shorebirds in different years; (2) to understand the shorebirds and their habitats in the wetland landscape patterns of extreme drought and a normal water year through a comparative analysis of landscape indices, shorebird diversity, and dominant species in the two areas; and (3) to analyze landscape indices, shorebird diversity, and dominant species in the two areas across different years. This study also aimed to analyze the response of the wetland landscape pattern, inhabited by shorebirds, to the extremely arid climate, (4) to quantify the correlation between dominant shorebird species and landscape characteristics, and to reveal shorebird responses to changes in landscape patterns. This study will provide essential information for the conservation of shorebirds and the management of their habitats.

## 2. Study Area

Poyang Lake (115°48′–116°44′ E, 28°25′–29°45′ N) is the largest freshwater lake in China, located in the northern part of Jiangxi Province. It is a significant watershed in the middle and lower reaches of the Yangtze River, bounded by the five water systems of the Gan, Fu, Xin, Rao, and Xiu Rivers and connected to the Yangtze River. The lake has a watershed area of 162,200 km^2^ [18]. Poyang Lake stretches approximately 170 km from north to south, with an average width of about 20 km from east to west. Its widest point reaches 50 km, and it has a water area of about 3583 km^2^ [18]. The water level of Poyang Lake exhibits seasonal dynamic changes, with the highest annual average water level in July. The water area fluctuates significantly between the abundant water period in summer and the dry period in winter. During the dry period from October to March each year, the water level becomes shallow, resulting in the formation of numerous shallow lakes of varying sizes. These features provide abundant food resources and habitats for wintering migratory birds [16,19,20,21,22].

**Figure 1:**
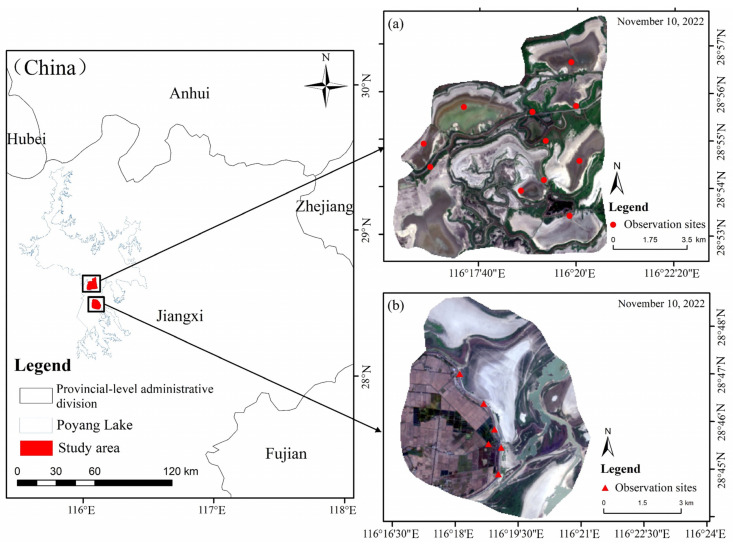
Location of Poyang Lake. (**a**) Location of Nanji Wetland National Nature Reserve, (**b**) location of Wuxing reclamation region.

Two areas of Poyang Lake were selected for this study: Nanji Wetland National Nature Reserve (hereinafter referred to as “Nanji Wetland”) and the Wuxing reclamation region (hereinafter referred to as “Wuxing”) (Figure 1). Nanji Wetland (116°10′~116°23′ E, 28°52′~29°06′ N) is located on the southern side of the main lake area of Poyang Lake, covering a total area of 33,300 km^2^, with a core area of 17,500 km^2^. It is classified as a permanent freshwater lake wetland and is a delta front area formed by the convergence of the North, Middle, and South branches of the Gan River into Poyang Lake. It also serves as an important node in the East Asia–Australia migratory bird flyway. The wetland lies at the junction of Nanchang, Jiujiang, and Shangrao. The main lake is a typical inland wetland dominated by islands, lakes, and grassy landforms and provides critical habitats for numerous wintering waterbirds [23]. Wuxing (116°11′–116°19′ E, 28°43′–28°48′ N) is located in the eastern part of the Nanchang High-technology Industrial Development Zone, covering an area of 52 km^2^. It is situated in the floodplain zone of Poyang Lake, characterized by fertile land. In addition to the natural habitats in Wuxing, it includes a large number of artificial habitats, such as rice paddies and lotus root ponds, which provide habitat for many wintering waterbirds [16,24].

## 3. Methods

### 3.1. Shorebird Survey and Data Processing

**Figure 2:**
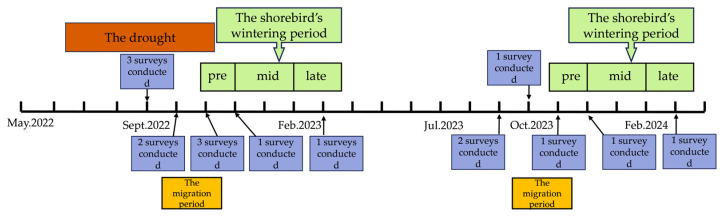
Timeline of the surveys for this study.

From September 2022 to April 2024, with the aid of binoculars (SWAROVSKI, 8×) and telescopes (SWAROVSKI, 20–60×), we ascertained that the area covered by the binoculars and telescopes had a radius of approximately 1 km. Three, two, three, and one survey were conducted in September, October, November, and December 2022. Two, one, one, and one survey were conducted in September, October, November, and December 2023, and one survey was conducted in March 2024 (Figure 2). Shorebird species and abundance were recorded using the direct count method in the sample area. The surveyed area of Nanji Wetland comprised eight lakes, namely Zhanbei Lake, San Lake, Chang Lake, San Ni Wan, Fengwei Lake, Baisha Lake, Nanshen Lake, and Beishen Lake, all of which are representative lakes of significant research value. The total area covered approximately 55.68 km^2^, with eleven sample points established. The survey area of Wuxing comprised an area extending 5 km from Hongjing Village along the embankment toward the conservation plot, encompassing approximately 30.94 km^2^ of natural habitat and nearly 15.29 km^2^ of artificial habitat. Due to the limited size of the lake, only six sample points were established along the embankment roads in the lake area to facilitate the observation of shorebirds in both natural habitats and cropland. All observation points were located at bird-watching platforms (approximately 2 m above the ground) and dyke observation areas. We ensured that all observation sample points were clearly visible and unobstructed, with no visible obstructions or impairments. Observation at each sample point lasted 10–20 min, until all shorebirds visible up to a distance of 1 km could be recorded. A longer time at one point was needed when the number of waterbirds was large.

The Berger–Parker dominance index (P) was employed to identify the dominant species of waterbirds (P ≥ 10%).

Berger–Parker Dominance Index: P = Ni/N(1)

where N_i_ represents the number of individuals of species i; and N represents the total number of individuals of all species in the community. We defined a species as a dominant species when P was larger than or equal to 10%.

The diversity of bird communities was calculated using Shannon’s diversity index, as shown in the following formula:

(2) $$H=-\sum_{i=1}^{S}p_{i}\ln(p_{i})$$

Evenness was measured using Pielou’s evenness index, as shown in Equation (3):

(3) J=H/ln(S)

where *H* represents the Shannon–Wiener diversity index; *p_i_* denotes the relative abundance of the *i*th species, typically calculated as the number of individuals (ni) of the species divided by the total number of individuals (N) of all species; *S* represents the total number of species; and ln refers to the natural logarithm. *J* represents the Pielou evenness index.

### 3.2. Classification of Habitat Types

Landsat images with a resolution of 3 m and cloud cover of less than 5% were selected for this study [15]. Landsat satellite data were acquired from the United States Geological Survey [25]. The satellite remote-sensing images were preprocessed using ENVI 5.3 (64-bit), and the Landsat satellite images underwent radiometric calibration, atmospheric correction, and geometric correction to obtain 8-phase remote-sensing images of the two study areas of Poyang Lake from 2022 to 2024. The object-oriented method was employed to categorize the wetland types of Poyang Lake, and land use types were classified into seven categories: deep water, shallow water, grassland, sandy beach, mudflat, pond, and cropland. Based on the multi-scale segmentation algorithm, the object-oriented method divides the remote-sensing image at a specific scale according to attribute features such as brightness, color, shape, and adjacency of pixels, merges pixel regions with similar attributes, divides them into multiple regions with the same attributes, and identifies and classifies the feature types based on pixel attribute thresholds [4,7]. Based on visual interpretation, various feature samples were selected for training to obtain thresholds for feature shape, texture, and spectral features (brightness, color). Based on the feature thresholds of each attribute type, the 8-phase remote-sensing images of wetlands in Poyang Lake were classified, and the classification accuracy was verified using visual interpretation, based on overall accuracy and the kappa coefficient. In this study, the overall accuracy of the 8-period land use classification results was 91%, and the kappa coefficient was 0.83.

In this study, the patch density (PD), maximum patch index (LPI), Shannon diversity index (SHDI), perimeter-area fractional dimension count (PAFRAC), and spreading degree index (CONTAG) were chosen to explore changes in landscape patterns at the landscape level (Table 1). Fragstats4 was employed to calculate landscape indices within the two regions of Poyang Lake at the landscape scale.

### 3.3. Correlation of Shorebird Diversity with Landscape Patterns

Data on the number of shorebird species, total number, number of dominant species, and landscape pattern-related indicators were entered into SPSS version 20. To avoid time-series autocorrelation, autocorrelation function (ACF) plots and partial autocorrelation function (PACF) plots of the time-series data were analyzed to detect autocorrelation between data points. Multiple comparisons were corrected using the FDR correction method. Spearman’s two-sided test was used to analyze the correlation between the relevant indicators and shorebirds, with statistical significance set at *p* < 0.05. A correlation of |*r*_s_| ≥ 0.7 was considered strong, 0.4 ≤ |*r*_s_| < 0.7 was considered moderate, and |*r*_s_| < 0.4 was considered weak.

## 4. Results

### 4.1. Area of Habitat Types During Extreme Drought and a Normal Water Year

**Figure 3:**
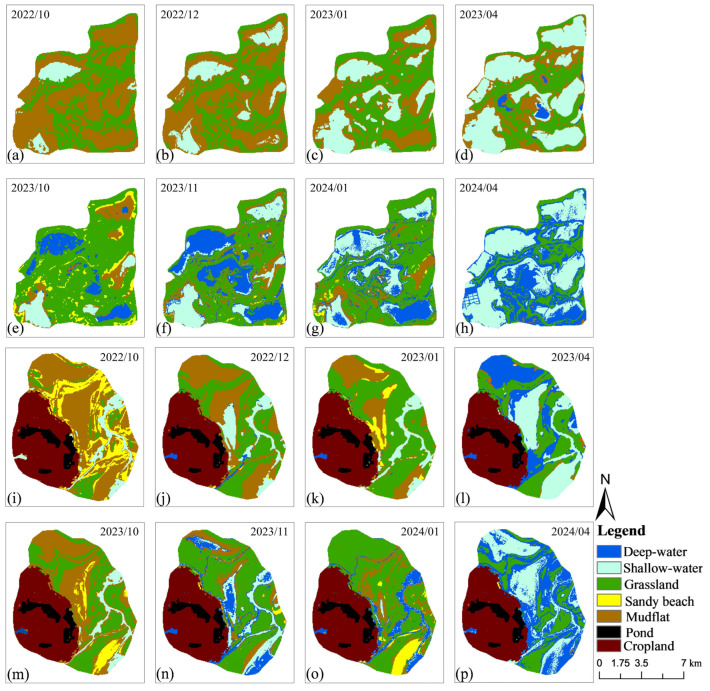
Composition of various habitat types during extreme drought (October 2022 to April 2023) and a normal water year (October 2023 to April 2024) in the Nanji Wetland (**a**–**h**) and Wuxing (**i**–**p**).

**Figure 4:**
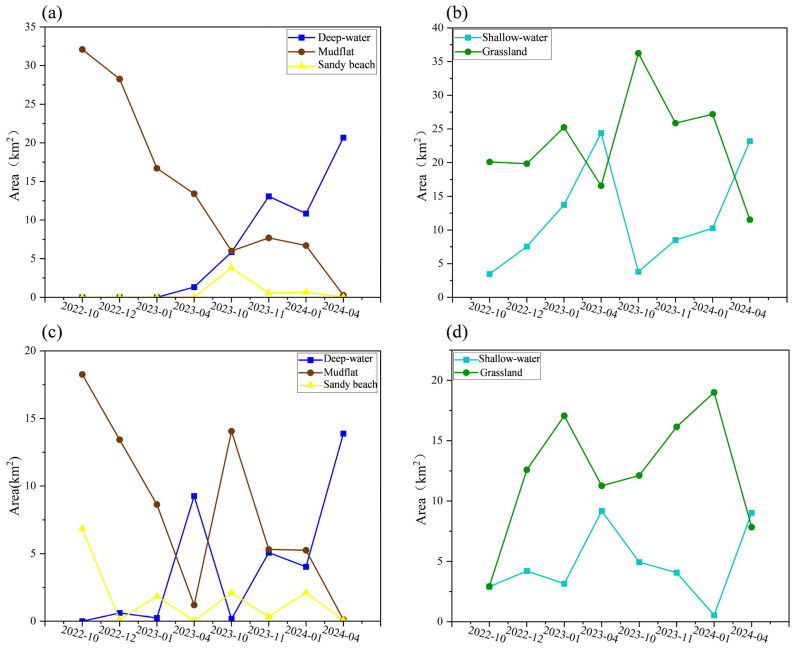
Changes in the area of various types of habitats in extreme drought and a normal water year in Nanji Wetland (**a**,**b**) and Wuxing (**c**,**d**) (km^2^).

The area of deep water throughout the overwintering period in the Nanji Wetland in the extreme drought year was greatly lower than that in the normal water year, showing an increasing trend in the whole overwintering period in both extreme drought and the normal water year (Figure 3). The area of mudflats in the extreme drought year was much larger than that in the normal water year, with a decreasing trend over time (Figure 4a). In the extreme drought year, the area of grassland was greatly lower than that in the normal water year in the pre-overwintering and mid-overwintering periods, and higher than that in the normal water year in the late-overwintering periods; the area of grassland showed a decreasing trend in the overwintering period and followed a similar decreasing trend in the normal water year. The area of shallow water in extreme drought and the normal water year was similar in all periods, and the trend of its area change was also similar, with both demonstrating an increasing trend in the overwintering period (Figure 4b). The area of deep water throughout the overwintering period in the Wuxing extreme drought year was greatly smaller than that in the normal water year, and the area of deep water demonstrated an increasing trend in both years. The area of mudflat was larger than that in the normal water year, and showed a decreasing trend in both years (Figure 4d). In the extreme drought year, the area of grassland was greatly lower than that in the normal water year, but the area during the late-overwintering period was larger than that in the normal water year, and the area of grassland in both years showed the same trend of increasing from the pre-overwintering to the mid-overwintering period, and decreasing in the late-overwintering period. In the extreme drought year, the area of shallow water was smaller in October than in the normal water year, remained relatively unchanged in December and April, and was larger in January than in the normal water year. In the extreme drought year, the area of shallow water remained relatively unchanged from the migration period to the mid- and late-wintering periods, while the area of shallow water in the normal water year decreased from the pre-overwintering to the mid-overwintering period, and then increased in the late-overwintering period (Figure 3e).

### 4.2. Changes in Landscape Indices During Extreme Drought and a Normal Water Year 

The patch density (PD), maximum patch index (LPI), and spreading degree index (CONTAG) were lower in all periods of the extreme drought year in Nanji Wetland compared to the normal water year. The perimeter-area fractional dimension (PAFRAC) was smaller in the extreme drought year than in the normal water year in January and April, with smaller changes observed in other periods. The Shannon diversity index (SHDI) was greater than that of the normal water year in April of the extreme drought year, and it was lower than in the normal water year during all other months (Table 2).

The patch density (PD) was higher in October of the extreme drought year than in the normal water year in Wuxing, but lower in all other months compared to the normal water year. The perimeter-area fractional dimension count (PAFRAC) was lower in all months of the extreme drought year compared to the normal water year. The maximum patch index (LPI), spreading degree index (CONTAG), and Shannon diversity index (SHDI) were similar to those of the normal water year (Table 3).

### 4.3. Composition of Shorebird Species in Extreme Drought and a Normal Water Year

**Figure 5:**
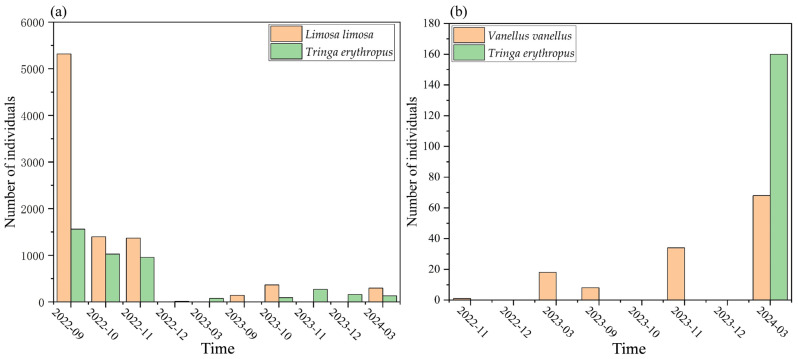
Temporal changes in the dominant species of shorebirds within the two areas of Nanji Wetland (**a**) and Wuxing (**b**).

In the extreme drought and normal water year, a total of 18,295 individuals from 21 shorebird species and 1836 individuals from 9 species of shorebirds were recorded in the Nanji Wetland, respectively. The dominant species in both years were the *Limosa limosa* and *Tringa erythropus* (Figure 5a), while *Vanellus vanellus*, with a population of 2330 in the extreme drought year, was also among the dominant species. The more abundant shorebirds in the Nanji Wetland typically exhibited significant fluctuations in their numbers throughout the survey period. For example, the number of *Limosa limosa* decreased from 5318 in September to 1399 in October and 1370 in November during the extreme drought year, while a sharp decline was also observed in the normal water year. The number of *Tringa erythropus* decreased continuously throughout the extreme drought year, from 1562 to 76, while their population remained relatively stable in the normal water year. In the extreme drought year, 773 *Tringa glareola* were recorded in September, whereas only 32 individuals were observed in the normal water year. In the extreme drought year, 80 *Calidris alpina* were recorded in September, with their numbers increasing to 1251 in November, while none were recorded in the normal water year (Table A1).

In the Wuxing extreme drought and normal water years, a total of 10 species of shorebirds were recorded, with 510 individuals in the extreme drought year and 15 species in the normal water year. The dominant species in both years were *Vanellus vanellus* and *Tringa erythropus* (Figure 5b), while *Recurvirostra avosetta* (110) and *Limosa limosa* (160) were dominant in the normal water year. The more abundant species exhibited significant fluctuations between years and months. For example, 280 *Vanellus vanellus* were recorded in November of the extreme drought year, but they were not observed in any other months. The numbers of these species declined sharply in the normal water year. *Limosa limosa* was not recorded during the extreme drought year, but 160 individuals were observed in March of the normal water year. The number of *Tringa erythropus* was greatly lower in the extreme drought year compared to the normal water year (Table A2).

### 4.4. Shorebird Diversity Parameters

The number of species, individuals, and diversity indices in Nanji Wetland were greatly higher during the extreme drought year compared to the normal water year, while the evenness index during the extreme drought year was lower than in the normal water year. The number of species, individuals, diversity index, and evenness index in Wuxing were lower during the extreme drought year compared to the normal water year (Table 4).

### 4.5. Correlation Between Shorebird and Landscape Indices

**Figure 6:**
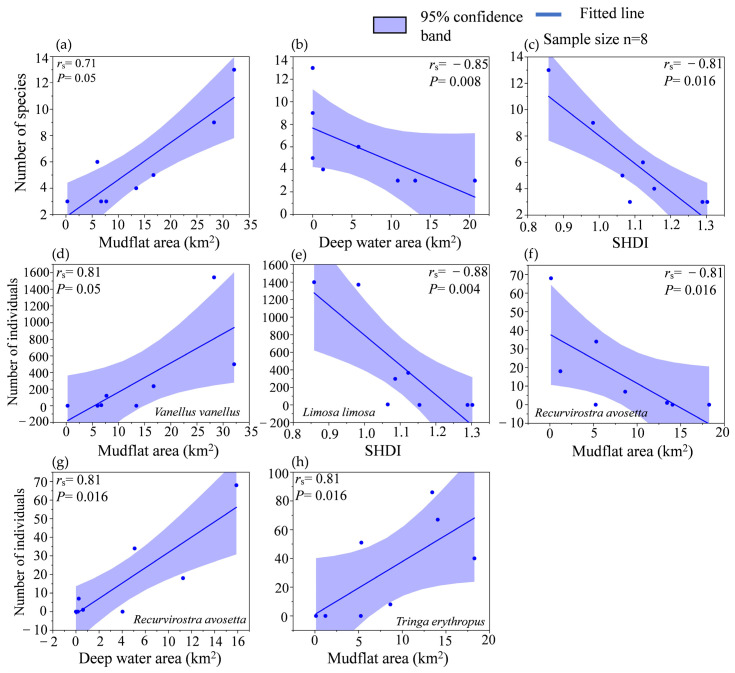
Correlation between shorebird, habitat area, and landscape indices in Nanji Wetland (**a**–**e**) and Wuxing (**f**–**h**).

The number of shorebird species in the Nanji Wetland showed a strong positive correlation with mudflat area (*r*_s_ = 0.71, *p* = 0.05) (Figure 6a) and a strong negative correlation with deep water area (*r*_s_ = −0.85, *p* = 0.008) (Figure 6b) and SHDI (*r*_s_ = −0.81, *p* = 0.016) (Figure 6c). *Vanellus vanellus* numbers showed a strong positive correlation with mudflat area (*r*_s_ = 0.81, *p* = 0.05) (Figure 6d). *Limosa limosa* showed a strong negative correlation with SHDI (*r*_s_ = −0.88, *p* = 0.004) (Figure 6e). Shorebird diversity parameters at Wuxing were not correlated with habitat indicators. *Recurvirostra avosetta* showed a strong negative correlation with mudflat area (*r*_s_ = −0.81, *p* = 0.016) (Figure 6f) and a strong positive correlation with deep water area (*r*_s_ = 0.81, *p* = 0.016) (Figure 6g); *Tringa erythropus* showed a strong positive correlation with mudflat area (*r*_s_ = 0.81, *p* = 0.016) (Figure 6h).

## 5. Discussion

### 5.1. Landscape Characteristics and Temporal Changes in Shorebirds

Shallow water and mudflats represent the most suitable habitats for shorebirds. The tarsal length of shorebirds ranges from 22 to 132 mm [26], and the depth of their habitat does not exceed the length of their tarsals, making shallower water more favorable for walking. Approximately 80% of benthic animals inhabit the sediment layer of 0–3 cm, and more than 95% inhabit the sediment layer of 0–6 cm [26,27,28]. The shorebirds’ beak is well-suited to probing food within these sediment layers. Deep water areas lack suitable food resources for shorebirds, and shorebirds foraging in these areas expend more energy [26,29]. Substrate resistance has been shown to significantly affect shorebird feeding behavior: the deeper a shorebird, the wider the range of prey it can reach, while energy expenditure during foraging is increased. Mudflats, which typically exhibit low substrate resistance, are considered the primary locations for shorebird foraging behavior [30]. In this study, it was found that both shallow and deep water areas were significantly reduced in the early stage of the extreme drought year, while mudflat areas were higher than in the normal water year in both study areas. The decrease in deep water area and the increase in mudflat area are generally favorable to shorebirds, while the decrease in shallow water area is unfavorable to them [28]. The number of shorebird species in the Nanji Wetland during the extreme drought year (21 species) was greatly higher than in the normal water year (9 species) and previous years (10 species) [23]. The number of shorebird species (10) in the extreme drought year at Wuxing was lower than the 15 species recorded in the normal water year. This was due to a significant reduction in the watershed area of Nanji Wetland during the extreme drought year, which promoted the concentration of food resources and attracted a temporary aggregation of shorebirds in the surrounding area. Although inland New South Wales, Australia has extensive lowland floodplains and wetlands, during drought events, the area of suitable inland habitat declines while the suitable coastal area increases, and shorebirds travel to coastal drought refuges by altering their migratory strategies [31,32], a result consistent with our findings. During extreme drought events, the original mudflats and shallow water areas of the lake hardened, benthic organisms experienced significant mortality, and the quality of the habitat declined dramatically. Shorebirds then migrated to new mudflats, and shallow-water areas of the lake with certain ecological functions that mitigated the effects of the extreme drought. Furthermore, the increase in mudflat area during the extreme drought year is also an important factor contributing to the higher number of shorebird species compared to previous years. During the extreme drought year, the number of shorebird species in Wuxing was considerably lower than in the normal water year, due to there being an insufficient shallow water area to meet the ecological needs of a large number of shorebirds. Large nature reserves serve as core components of biodiversity conservation and help mitigate biodiversity loss under extreme climate conditions [33]. Therefore, the extensive area of Nanji Wetland plays a decisive role in protecting shorebirds and other waterbirds and can serve as a refuge for these species in extreme environments. This finding is consistent with the results in the East Dongting Lake, Hunan Province [34], which showed that the number of waterbirds in the core area of East Dongting Lake also remained high during the extreme drought year. This is because, on the one hand, the water level in large, protected areas or core areas is artificially monitored and dynamically regulated, allowing shorebirds to survive in certain areas and adapt to extreme climatic conditions, thereby preventing the excessive loss of water in the wetland, which could lead to the death of large numbers of waterbirds. On the other hand, large, protected areas can effectively control human interference, such as reducing agricultural land use and urbanization. Low-intensity human disturbance is more likely to attract the aggregation of waterbirds. During the middle and late stages, the water level of the two areas of Poyang Lake was restored due to rainfall and water sources from the Yangtze River Basin [35]. However, shorebirds primarily feed on benthic animals, aquatic insects, and other animal-based food sources. The death of benthic animals caused by the early drought, along with the over-consumption of benthic animals under a high concentration of shorebirds during the early period, prevented the recovery of benthic and other animals after the water area increased during the late overwintering period. As a result, the number of *Tringa erythropus* in the mid and late periods of the Nanji Wetland was much lower than that during the normal water year. Due to the increase in water levels during the late stages, some cropland and root ponds began to store water, providing foraging habitats for some shorebirds such as *Recurvirostra avosetta*, *Vanellus vanellus*, and *Calidris alpin*. As a result, the number of shorebirds during the mid to late stage of the extreme drought year was higher than in the early stage. Additionally, the number of *Limosa limosa* and *Recurvirostra avosetta* in the mid and late stages of the normal water year exceeded that in the early stage, due to the restoration of some suitable habitats and the functioning of artificial habitats.

### 5.2. Response of Shorebirds to Habitat Types and Landscape Patterns

This study indicates a strong positive correlation between the area of mudflats and the number of shorebirds, particularly the population sizes of *Vanellus vanellus* and *Tringa erythropus* populations, with a strong negative correlation observed only for *Recurvirostra avosetta* populations. Different areas of the mudflats create diversified ecological niches [36,37], allowing various shorebirds to select suitable microhabitats based on their foraging habits and ecological needs [38,39], which facilitates the coexistence of different shorebirds. The mudflats expose a large number of benthic organisms, and the abundant food resources attract various shorebirds to forage and rest, thereby increasing both the overall number of shorebirds and the population of specific shorebirds. Field observations revealed that *Vanellus vanellus* primarily feeds on aquatic insects in mudflats. *Tringa erythropus* is widely distributed in Poyang Lake, and typically gathers in large groups, ranging from dozens to thousands, to forage in shallow water and mudflat areas [40,41]. The drought increased the area of mudflats and the insect density in inland lakes, such as *Chironomus* and *Psychoda* larvae, provided larger feeding and resting sites for *Tringa erythropus* [42]. Furthermore, the mudflats and surrounding shallow waters became richer in food resources, which attracted more *Tringa erythropus*, helping them cope with the extreme climate. Therefore, a strong positive correlation exists between the presence of these two species and the area of mudflats. The negative correlation between mudflat area and *Recurvirostra avosetta* may be attributed to intense interspecific competition in dry environments, which reduces the *Recurvirostra avosetta* population. Additionally, *Recurvirostra avosetta*, being a relatively larger bird, prefers to feed in deeper shallow waters and paddy fields. An increase in mudflat areas may reduce their foraging space, leading to a negative correlation between the two. The area of mudflats was negatively correlated with *Recurvirostra avosetta*. *Recurvirostra avosetta* is a relatively large species, and they prefer to forage in deeper, shallow water and paddy fields. The increase in mudflat area may compress the area of some shallow water, thereby decreasing the suitability of *Recurvirostra avosetta* habitats. The SHDI was strongly and negatively correlated with the number of shorebird species and the number of *Limosa limosa* individuals. The SHDI indicates the diversity of various habitat types; the higher the SHDI, the more fragmented the habitats became, resulting in small, scattered patches [39]. As a result, the landscape shapes become more complex and irregular, and resources become unevenly distributed in space, leading to greater difficulties in moving and foraging [43]. A larger SHDI decreases connectivity between sub-lakes. Moreover, shorebirds are among the most sensitive waterbirds. Highly sensitive waterbirds choose habitats with concentrated patches and high connectivity, such as storks, cranes, and shorebirds. Less sensitive waterbirds choose habitats with fragmented patches and diverse types, such as geese, ducks, and herons [44]. Therefore, the number of shorebird species is negatively correlated with the SHDI. *Limosa limosa* individuals are large and prefer to gather in large groups to forage [40]. Areas with higher SHDIs are not conducive to large groups of *Limosa limosa* foraging and therefore exhibit a strong negative correlation. Our findings have to be further tested for a long-term period in the future.

## 6. Conclusions

The area of deep water in Nanji Wetland and Wuxing during an extreme drought year is smaller than in a normal water year. The area of mudflats is larger, while the area of grassland is smaller in the early and middle stages of the extreme drought year. The fluctuation in the area of shallow water in both locations is relatively small. The landscape indices of Nanji Wetland were more variable, with most indices being lower during the extreme drought year. Most landscape indices for Wuxing showed little variation across years. The increase in mudflat area and the decrease in shallow water area concentrated food resources. Consequently, the number of species and individuals during the extreme drought year in Nanji Wetland was higher than in the normal water year, suggesting that the large, protected area serves as a refuge for many shorebirds. Mudflats are critical habitats for shorebirds, with their area showing a strong positive correlation with the number of dominant species, as well as the populations of *Vanellus vanellus* and *Tringa erythropus.* The SHDI exhibited a strong negative correlation with the number of shorebird species and the population of *Limosa limosa*. Therefore, increasing the mudflat area is beneficial for maintaining shorebird diversity, while decreasing the SHDI can also enhance shorebird diversity. We should maintain the area of mudflats. However, implementing these strategies will encounter challenges due to agriculture, urbanization, and policy frameworks. This study examined the response of shorebirds to an extreme climate at a small scale, which improved the accuracy of the results. However, the response of shorebird diversity to these ecological factors across Poyang Lake as a whole needs further verification. In the future, a larger-scale landscape analysis and diversity survey should be conducted for a more objective and comprehensive evaluation of waterbird diversity conservation.

## Figures and Tables

**Table 1:** Description of landscape pattern indices.

Landscape Index	Clarification
Patch density (PD)	PD = (NP/A) where A represents the area of the landscape or a specific type of patch; PD denotes the number of patches per unit area; and NP represents the number of patches, which reflects the degree of landscape fragmentation. The larger the PD value, the higher the degree of landscape fragmentation.
Maximum patch index (LPI)	$LPI=\frac{a_{\max}}{A}$ (0 < *LPI* < 100%) where a_max_ represents the maximum patch area of the landscape or a specific patch type, and *A* represents the total area of the landscape or a specific patch type; *LPI* reflects the dominant patch type in the landscape and quantifies the proportion of the largest contiguous patch relative to the total landscape area. It indicates the degree of continuity and fragmentation of the landscape. A value of 0% signifies the absence of large contiguous patches, while 100% indicates that the entire landscape consists of a single large patch.
Shannon diversity index (SHDI)	$SHDI=-\sum_{i=1}^{n}\left(P_{i}\ln P_{i}\right)$ (0 ≤ *SHDI*) where P_i_ represents the proportion of the patch area of category i relative to the total area of the landscape; and n denotes the number of patch types in the landscape. *SHDI* reflects landscape heterogeneity; a value of 0 for *SHDI* indicates that the landscape consists of a single patch type, and an increase in *SHDI* indicates a greater level of landscape heterogeneity.
Perimeter-area fractional dimension count (PAFRAC)	$PAFRAC=\frac{\frac{2}{\left[n_{i}\sum_{j=1}^{n}\left(\ln p_{ij}\times \ln p_{ij}\right)\right]-\left[\left(\sum_{j=1}^{n}\ln p_{ij}\right)\left(\sum_{j=1}^{n}\ln a_{ij}\right)\right]}}{\left(n_{i}\sum_{j=1}^{n}\ln{p_{ij}}^{2}\right)-{\left(\sum_{j=1}^{n}\ln p_{ij}\right)}^{2}}$ (1 ≤ *PAFRAC* ≤ 2) where *p_ij_* represents the perimeter of the jth patch of the ith patch type; a_ij_ represents the area of the *j*th patch of the ith patch type; *n_i_* represents the number of patches of the ith patch type. The closer PAFRAC is to 1, the more regular the shape of the patch, and the closer PAFRAC is to 2, the more complex the shape becomes.
Spreading degree index (CONTAG)	$CONTAG=\left[1+\frac{\sum_{i=1}^{m}\sum_{i=1}^{m}\left[\left(p_{i}\right)\left(\frac{g_{ik}}{\sum_{k=1}^{m}g_{ik}}\right)\right]\left[\ln\left(p_{i}\right)\left(\frac{g_{ik}}{\sum_{k=1}^{m}g_{ik}}\right)\right]}{2\ln\left(m\right)}\right]\times 100$ (0 < *CONTAG* ≤ 100) where *P_i_* denotes the percentage of area occupied by patch type *i*; *g_ik_* denotes the number of adjacent patches of types i and k; and m denotes the number of patch types in the landscape. CONTAG reflects the degree of aggregation or the trend of extension of patches in the landscape. A high CONTAG indicates that the landscape consists of dominant patch types with better connectivity, while a low index suggests that the landscape comprises a wide range of patch types with poorer connectivity.

**Table 2:** Landscape indices for extreme drought and a normal water year in Nanji Wetland.

Year	Time	PD	LPI	PAFRAC	CONTAG	SHDI
Extreme drought	2022-10	3.718	27.151	1.342	47.740	0.859
	2022-12	8.049	23.356	1.420	38.032	0.983
	2023-01	3.448	43.961	1.324	38.782	1.065
	2023-04	3.663	19.541	1.331	47.465	1.154
Normal water	2023-10	6.555	63.885	1.314	55.855	1.122
	2023-11	27.440	41.049	1.495	42.723	1.302
	2024-01	32.558	45.710	1.512	40.820	1.289
	2024-04	9.967	34.928	1.427	52.393	1.086

PD: patch density; LPI: maximum patch index; PAFRAC: perimeter-area fractional dimension; CONTAG: spreading degree index; SHDI: Shannon diversity index.

**Table 3:** Landscape indices for extreme drought and a normal water year in Wuxing.

Year	Time	PD	LPI	PAFRAC	CONTAG	SHDI
Extreme drought	2022-10	6.922	27.847	1.378	47.663	1.509
	2022-12	5.018	27.847	1.288	53.709	1.513
	2023-01	5.797	27.847	1.274	53.741	1.531
	2023-04	6.619	27.847	1.297	51.260	1.598
Normal water	2023-10	5.5599	27.818	1.383	51.380	1.542
	2023-11	19.661	27.847	1.466	47.178	1.619
	2024-01	21.067	27.847	1.465	49.517	1.529
	2024-04	11.010	27.847	1.426	50.113	1.518

PD: patch density; LPI: maximum patch index; PAFRAC: perimeter-area fractional dimension; CONTAG: spreading degree index; SHDI: Shannon diversity index.

**Table 4:** Overwintering diversity parameters of Nanji Wetland and Wuxing in extreme drought and a normal water year.

	Nanji Wetland		Wuxing	
Diversity Parameter	Extreme Drought	Normal Water	Extreme Drought	Normal Water
Number of species	21	9	10	15
Number of individuals	18295	1836	510	784
H	2.53	2.03	1.80	2.95
J	0.57	0.64	0.54	0.76

## Data Availability

The data presented in this study are available upon request from the corresponding author. The data are not publicly available due to the constraint of consent.

## Appendix A

**Table A1:** Composition of shorebirds in extreme drought and a normal water year in Nanji Wetland.

	2022–2023					2023–2024				
Months	9	10	11	12	3	9	10	11	12	3
Species										
I CHARADRIIFORMES										
(I) Recurvirostridae										
1. *Himantopus Himantopus*	64					22	3			
2. *Recurvirostra avosetta*	57	26	288	22	140			33	2	
(II) Charadriidae										
3. *Vanellus vanellus*	52	499	1544	235				120	7	
4. *Vanellus cinereus*	293	3				19				
5. *Charadrius dubius*	255	111								
6. *Charadrius alexandrines*	92	20	2	2						
(III) Scolopacidae										
7. *Gallinago megala*	1									
8. *Gallinago gallinago*	132	4								
9. *Limosa limosa*	5318	1399	1370			140	366			297
10. *Numenius arquata*	1562	1028	956	14	76		94	270	159	132
11. *Tringa erythropus*	134	49	2				30			
12. *Tringa stagnatilis*	162	183	7	2	4	22	83			2
13. *Tringa nebularis*	773	16		1		32	3			
14. *Tringa glareola*				1						
15. *Actitis hypoleucos*	11									
16. *Calidris tenuirostris*	2									
17. *Calidris canutus*	1	1								
18. *Calidris ruficollis*	21		5	2						
19. *Calidris temminckii*	2	6								
20. *Calidris pugnax*	80		1251	10						
21. *Calidris alpina*			2		2					

**Table A2:** Composition of shorebirds under extreme drought and a normal water year in Wuxing.

	2022–2023					2023–2024				
Months	9	10	11	12	3	9	10	11	12	3
Species										
I CHARADRIIFORMES										
(I) Recurvirostridae										
1. *Himantopus Himantopus*						4				
2. *Recurvirostra avosetta*			1		18	8		34		68
(II) Charadriidae										
3. *Vanellus vanellus*			280			35		80	11	
4. *Vanellus cinereus*	3									
5. *Charadrius dubius*	1					30	3			
6. *Charadrius alexandrines*						8	1			
(III) Scolopacidae										
7. *Gallinago gallinago*	5									
8. *Limosa limosa*										160
9. *Tringa erythropus*						2				
10. *Tringa stagnatilis*		40	1	86		42	67	51		
11. *Tringa nebularis*						2				
12. *Tringa ochropus*	2	7				41	2	2		
13. *Tringa glareola*	4					129				
14. *Actitis hypoleucos*			2			1				
15. *Calidris canutus*						1				
16. *Calidris temminckii*						2				
17. *Calidris pugnax*										
18. *Calidris alpina*				60						
